# Supplementary material for: A Machine Learning Approach to Screen for Otitis Media Using Digital Otoscope Images Labelled by an Expert Panel
Source: Diagnostics (Basel). 2022 May 25;12(6):1318. doi: 10.3390/diagnostics12061318 (PMC9222011; doi:10.3390/diagnostics12061318)
Supplement: Supplementary file 1 [file diagnostics-12-01318-s001.zip › diagnostics-1701469-supplementary.pdf]

Definition of diagnoses

Normal – pearly gray and transparent TM in fairly normal position with coherent light reflex and a clearly visible short process of the malleus. The TM has a “ground-glass” appearance.

OME - Opaque TM or a visible air–fluid interface behind a transparent TM. TM can be in a normal or retracted position.

AOM - Mild - to moderate or severe - bulging of a cloudy (opaque) tympanic membrane (TM) and/or a chagrinated and opaque TM. A discoloration (yellow/white) and/or intense erythema of the TM may also be present.

CSOM - A non-intact TM (perforation) without non-purulent ear discharge. Ears with purulent discharge are not included in the study but slightly wet and non-purulent ears may be shown

Wax – a yellowish to brown/black waxy substance obstructing the entire ear canal

NPD – Not possible to determine due to poor image quality or restricted view of the TM (caused by wax, other obstructing material or poor angle of image). Could also be used if a diagnosis occurs that is not included in the study.
